# Effects of Poison in the Treatment of Certain Affections

**Published:** 1855-11

**Authors:** 


*Effects of Position in the Treatment of certain Gastric and Enteric affections.* By Dr. COALE.

At a meeting of the "Boston Society for Medical Improvement," Dr. Coale remarked "that the late freqency of cholera morbus and other similar affections, had given him an opportunity of testing, to a considerable extent, the efficacy of a certain practice of his, based upon observation made some time since, but which he felt wanted confirmation before suggesting it generally. He is convinced, from actual experiment, that persons affected with irritability of the stomach are much less liable to vomit if they lie on the right side than when they recline in any other position—particularly on the left side. The explanation is evident. While lying on the righ side, any contraction of the stomach need not much affect its solid contents; but, when lying on the left side, the contents are in the neighborhood of the cardiac orifice, and any contraction of the organ will force them more or less through this opening into the œsophagus ; thus, the difference between the two cases will be a simple eructation in the first, and vomiting in the second. This, Dr. C. has now tested in very many cases : and by many experiments in some of them, varying the position to the increase or diminution of the nausea and vomiting. It may be urged in objection to the explanation, that a contraction of the stomach that would force the contents through the cardiac orifice, would produce vomiting at any rate. But the difference is this: the same amount of contraction which, when the patient lies on the right side, throws off gas merely, when he is on the other may force a small portion of solid or fluid matter into the œsophagus, when reflex action is at once excited and the whole stomach stimulated into action.

"In treatment of cases of flatulence, and of what is commonly called 'cramp colic,' Dr. C. has found reclining on the right side beneficial. It lessens the vomiting—as first said—a frequent attendant in these cases ; but, besides this, it gives a more ready escape to gas contained in the transverse colon. For example, suppose the trouble is a spasm, confining gas in the transverse or ascending colon, were the patient on the left side, and a relaxation of the spasm to occur, the gas is still kept behind the affected spot, for the distended intestine is not liable to take upon itself sufficient action to expel it. But, if the patient be on the right side, the gas then ascends and passes on to an unaffected part of the intestine, by which its escape is facilitated."—*American Quarterly Journal of Medical Science.*

